# The effects of China’s urban basic medical insurance schemes on the equity of health service utilisation: evidence from Shaanxi Province

**DOI:** 10.1186/1475-9276-13-23

**Published:** 2014-03-09

**Authors:** Zhongliang Zhou, Liang Zhu, Zhiying Zhou, Zhengya Li, Jianmin Gao, Gang Chen

**Affiliations:** 1 School of Public Policy and Administration, Xi’an Jiaotong University, Xi’an, China; 2 Department of Medical Education, Fourth Military Medical University, Xi’an, China; 3 Institute of Health Administration and Policy, Xi’an Jiaotong University, Xi’an, China; 4 School of Foreign Studies, Xi’an University, Xi’an, China; 5 Flinders Health Economics Group, School of Medicine, Flinders University, 216 Daws Road, Daw Park, Adelaide, SA 5041, Australia

## Abstract

**Introduction:**

In order to alleviate the problem of “Kan Bing Nan, Kan Bing Gui” (medical treatment is difficult to access and expensive) and improve the equity of health service utilisation for urban residents in China, the Urban Employee Basic Medical Insurance scheme (UEBMI) and Urban Resident Basic Medical Insurance scheme (URBMI) were established in 1999 and 2007, respectively. This study aims to analyse the effects of UEBMI and URBMI on the equity of outpatient and inpatient utilisation in Shaanxi Province, China.

**Methods:**

Using the data from the fourth National Health Services Survey in Shaanxi Province, the method of Propensity Score Matching was employed to generate comparable samples between the insured and uninsured residents, through a one-to-one match algorithm. Next, based on the matched data, the method of decomposition of the concentration index was employed to compare the horizontal inequity indexes of health service utilisation between the UEBMI/URBMI insured and the matched uninsured residents.

**Results:**

For the UEBMI insured and matched uninsured residents, the horizontal inequity indexes of outpatient visits are 0.1256 and -0.0511 respectively, and the horizontal inequity indexes of inpatient visits are 0.1222 and 0.2746 respectively. Meanwhile, the horizontal inequity indexes of outpatient visits are -0.1593 and 0.0967 for the URBMI insured and matched uninsured residents, and the horizontal inequity indexes of inpatient visits are 0.1931 and 0.3199 respectively.

**Conclusions:**

The implementation of UEBMI increased the pro-rich inequity of outpatient utilisation (rich people utilise outpatient facilities more than the poor people) and the implementation of URBMI increased the pro-poor inequity of outpatient utilisation. Both of these two health insurance schemes reduced the pro-rich inequity of inpatient utilisation.

## Introduction

Health service equity is an important goal pursued by health policy makers and health systems [1]. Since the early 21st century, increasingly more countries have shifted focus on formulating health policy to reducing the health service inequality; China is no exception. International experiences suggest that a well-established health insurance system could increase the health service utilisation for low-income residents and further improve the health service equity for the poor [2,3]. However, since the end of 1970s, along with the market economic reform, the near universal medical insurance schemes had largely collapsed in China. Only 38% of urban people were covered by any kind of medical insurance scheme in 1998 [4]. In the meantime, the proportion of patients who had forgone outpatient utilisation and hospitalisation reached 49.7% and 29.5%, respectively [4]. Among them, the majority of patients came from low-income families.

In order to alleviate the problem of “Kan Bing Nan, Kan Bing Gui” (too expensive to see doctors and too difficult to access doctors) and to improve the equity of health service utilisation for urban residents, the Chinese state government established two basic medical insurance schemes: the Urban Employee Basic Medical Insurance scheme (UEBMI) and the Urban Resident Basic Medical Insurance scheme (URBMI), to cover the urban residents in 1999 and 2007, respectively [5,6]. The UEBMI covers employees and retirees from enterprises and public institutions, whilst the other urban residents, including the unemployed, children and students, are covered by the URBMI [7]. By the end of 2012, more than 37% and 33% people were covered by UEBMI and URBMI across the country [8]. With the expansion of the basic medical insurance coverage in urban China, increasingly more patients have benefited from the Schemes. A brief introduction about the UEBMI and URBMI from the sample province is introduced below.

The UEBMI and URBMI were implemented in Shaanxi Province in the years 1999 and 2007, respectively. By the end of 2012, there were 5.47 million residents and 5.71 million residents covered by UEBMI and URBMI, which accounts for 29.2% and 30.4% of the total population in Shaanxi Province, respectively [9]. The detailed financing and reimbursement policies and benefit packages of the two urban basic medical insurance schemes from the sample province in 2008 are summarised in Table 1.

**Table 1:** Comparison of UEBMI and URBMI policies in Shaanxi Province in 2008

	**UEBMI**	**URBMI**
Year of launch	1999	2007
Enrolment unit/type	Individual/Mandatory	Individual/Voluntary
Sources of revenues	The employer contribute 6% of the employee’ salary whilst the employee contribute 2%; the retired are exempt from premium contribution	200 to 350RMB per person per year for residents (including government contributions)
Accounts	Medical Savings Account (including employee contributions and 30% of employer contributions) for outpatient utilisation; Social Pooling Account (70% of employer contributions) for inpatient utilisation and critical (i.e. chronic or fatal disease) outpatient utilisation	Social Pooling Account (all funds) for inpatient utilisation and critical (i.e. chronic or fatal disease) outpatient utilisation
Benefit package^†^ of Social Pooling Account	*INPATIENT & CRITICAL OUTPATIENT*	*CRITICAL OUTPATIENT*
	• Deductible:	• Deductible:
	6%-10% of local employees’ annual average wage	0-350 RMB
		• Reimbursement rate:
	• Reimbursement rate:	50%-70%
	80%-95%	• Ceiling:
	• Ceiling:	2,000-40,000 RMB
	4 times of local employees’ annual average wage	*INPATIENT*
		• Deductible:
		550-800, 400–600, 300–400 and 150–300 RMB for tertiary, secondary, primary, and community medical institutes, respectively
		• Reimbursement rate:
		40%-75% for adults; 45%-85% for students and residents under 18 and not attending school
		• Ceiling:
		24,000-35,000 RMB for adults; 30,000-60,000 for students and residents under 18 and not attending school
Payment method	Fee-for-service	Fee-for-service

Data resource: Statistical Bulletin of National Economy and Social Development in Shaanxi Province of the Year 2012, Xi’an municipal, and Shaanxi provincial policy documents.

^†^Vary across cities/counties and medical institute levels.

Theoretically, being insured, the equity of health service utilisation of the people from low-income families could be improved through the increased health service access. So far the evidence is mixed with regard to this hypothesis. By employing the indirect standardisation method for health service utilisation, Schneider et al. [2] analysed the relationship between being enrolled in a community-based health insurance scheme and the equity of basic health service utilisation in Rwanda. Their results show that the equity of health service utilisation for the insured people was better than the uninsured people. Using data from Taiwan, Lu et al. shows that the low-income people’s health service utilisation was improved after enrolling in the National Health Insurance scheme, including utilisation of western medicine, dental visits, hospitalisation utilisation and emergency visits [3]. Not all the studies found significant effects of medical insurance. Using the data from Indonesia, Hidayat found that the effects of mandatory health insurance on equity in access to outpatient care could be ignored [10].

There are very few studies investigating the effects of urban basic health insurance schemes on the equity of health service utilisation in China. Regarding the effects of UEBMI on health utilisation equity, there are only two related studies, with inconclusive results. Employing the longitudinal survey data from 1994 to 1996 in Zhenjiang city, Liu and his colleagues show that the equity of outpatient service utilisation was improved largely by the pilot UEBMI [11]. On the contrary, using more recent survey data from Shanghai city, Dong found that the equity of outpatient utilisation was reduced by the Medical Savings Account (MSA) in UEBMI [12]. The effects of URBMI on health utilisation equity, after comparing the equity of inpatient service utilisation before and after the implementation of URBMI, Guan et al. found that the effect of URBMI on improving the equity of inpatient utilisation is limited [13]. Both Liu et al. and Dong studied the effects of UEBMI on the equity of outpatient utilisation but omit inpatient service utilisation, whilst Guan et al. only studied the effect of URBMI on equity of inpatient utilisation but leaves outpatient utilisation unanalysed. So far only two studies have investigated the effects of these two basic medical insurance schemes in the same framework and studied both outpatient and inpatient utilisation in urban China. Wang et al. [14] found that both of UEBMI and URBMI were pro-rich for outpatient utilisation and inpatient utilisation. Gao et al. [15] found that compared with those insured by URBMI, the UEBMI enrollees’ outpatient utilisation equity is worse, but inpatient utilisation equity is better.

This study aims to investigate the effects of UEBMI and URBMI on the equity of both outpatient and inpatient utilisation using cross-sectional household survey data. The results of this study could shed light on the future policy implementation in Shaanxi Province, China.

## Methods

### Ethics

The study protocol was reviewed and approved by the Ethics Committee of Xi’an Jiaotong University School of Medicine. The data of this study was drawn from the 4th National Health Services Survey (NHSS) and an extended sample in Shaanxi Province surveyed in 2008. The NHSS was organised and directed by the Center for Health Statistics and Information of the Ministry of Health of China. This study only used the survey data from Shaanxi Province, through the Shaanxi Health and Family Planning Commission (http://www.sxhealth.gov.cn). Combing the extended sample, there were 44 sample counties included in the dataset that were representative of Shaanxi Province (see Additional file 1: Table S1 for sample representative test statistics). The data was anonymised when we accessed it and no study subject was directly approached.

### Data

Using the multi-stage stratified random sampling method, 7948 individuals (from 2721 households) were surveyed in the urban Shaanxi Province from June 15 to July 10, 2008. Since this study focused on the residents aged 15 years or older, the valid sample size is 5072 individuals, of whom 2801 residents were covered by UEBMI, 822 residents covered by URBMI and 1449 did not purchase either of the two insurance schemes.

### Indicators for measuring health service utilisation

Outpatient visits in the last two weeks and inpatient visits in the past year were selected as the indicators to measure the outpatient service utilisation and the inpatient service utilisation respectively in this study.

### Propensity score matching (PSM)

As there is a potential adverse selection issue between the insured and uninsured residents, in order to control the observable characteristics of respondents, the PSM method developed by Rosenbaum and Rubin [16] is used to match the insured and uninsured residents before calculating the equities of health utilisation. In the treatment effect evaluation study with observational data, selection bias often causes difficulty in establishing a casual relationship, and comparisons between treatment and control are hampered by the high dimensionality of the observed characteristics. In this circumstance, PSM does well to yield unbiased estimates of the treatment impact when a large number of control variables are available [17].

PSM method includes several candidate mapping algorithms, such as one-to-one matching, one-to-many matching and many-to-many matching [18]. The one-to-one matching algorithm is adopted in this study as we will later adopt the matched data in the regression analysis to calculate the equity. The logistic regression model was used to generate the propensity score. The independent variable is a dummy variable indicating whether or not an individual was covered by a medical insurance scheme (i.e. UEBMI or URBMI). The independent variables are those observable socio-demographic characteristics that are relevant for the insurance purchasing decision, including economic level, gender, age, self-reported health status, education, employment status, living region, whether or not purchased commercial health insurance, and household size. Economic level was measured by self-reported annual personal expenditure (calculated as annual household expenditure divided by household size). Self-reported health status for the day of the survey was the VAS score from the Chinese version of the EQ-5D instrument. It is a self-ranked score between 0 (the worst imaginable health status) and 100 (the best imaginable health status). Summary statistics for independent variables are shown in Table 2.

**Table 2:** Summary statistics for independent variables (mean/percent)

	**UEBMI**	**URBMI**	**Uninsured**
Annual personal expenditure (RMB, 2008)	8862	7086	6866
Gender			
Male^†^	54.6	42.1	42.5
Female	45.4	57.9	57.5
Age (years)			
15-44^†^	32.1	60.6	61.2
45-59	41.3	22.9	22.3
60+	26.5	16.4	16.5
Health status	77.8	81.4	81.8
Marital status			
Single^†^	4.6	25.2	26.0
Married	87.6	64.0	63.6
Others	7.8	10.8	10.5
Education status			
Illiterate^†^	3.1	7.9	8.5
Primary school	8.8	8.3	10.3
Junior middle school	26.1	28.6	32.8
Senior middle school	39.1	42.7	34.2
Diploma	14.3	8.4	7.2
College degree or above	8.5	4.1	7.0
Employment status			
Employed^†^	50	28	20.9
Retired	42.1	7.4	7.4
Student	0.1	13.5	16.0
Unemployed	7.8	51.1	55.8
Region			
Shanbei^†^	6.3	4.6	9.0
Guanzhong	87.5	95.3	80.5
Shannan	6.2	0.1	10.6
Commercial health insurance			
Insured	6.0	5.1	4.2
Uninsured^†^	94.0	94.9	95.8
Household population	3.00	3.08	3.15

^†^Reference levels in the Logistic regression.

Whether the matching procedure is able to balance the distribution of the control variables in the insured and uninsured residents should be checked. Standardised bias and two-sample *t*-test were used to assess the matching quality [19]. Standardised bias, suggested by Rosenbaum and Rubin [19], is to assess the distance in marginal distributions of the variables. If standardised bias is below 5% after matching, the matching result is seen as sufficient. The two-sample *t*-test is to check if there are significant differences in covariate means for the insured residents and uninsured residents. If there are no significant differences of covariates between the insured and uninsured residents after matching, the matching result is seen as a success.

### Methods of measuring health utilisation equity

A concentration index was employed to measure the income-related inequality of health service utilisation. Decomposition of the concentration index was employed to measure the horizontal inequity of health service utilisation.

The concentration index (CI) could quantify the degree of income-related inequality in health service utilisation [20-22]. The concentration index is zero if there is no income-related inequality of health utilisation. If the concentration index takes on a positive (negative) value, there is a pro-rich (pro-poor) inequality in health utilisation. The concentration index is calculated using Equation 1 [23-25]:

(1) $$C=\frac{2}{\mu }\mathrm{cov}\;\left(y,r\right)$$

where *C* is concentration index, *y* is health service utilisation, *μ* is the mean of health service utilisation, *r* is the fractional rank of income, ranging from 0 to 1. The rank of the *i* individual is: *r*_*i*_ = *i/N*, in which *N* is the number of individuals.

Decomposition of the concentration index method is adopted to decompose the measured degree of health utilisation inequality into the contributions of need variables and other control variables [26-28]. The horizontal inequity (HI) index of health service utilisation is computed by subtracting the contribution of need variables from the concentration index of health service utilisation, which is used to measure the equity of health service utilisation [29]. Healthcare need reflects the residents’ need for health service utilisation, which was measured by gender, age, self-reported health status, illness in the last two weeks and chronic disease following the literature [30].

As both outpatient visits and inpatient visits are count variables, negative binomial models were used to decompose the inequality of health service utilisation, in which the dependent variables are outpatient visits in the last two weeks, and inpatient visits in the last year, respectively [25]. Apart from need variables, other control variables in the model include self-reported annual personal expenditure, marital status, commercial health insurance, household population and distance to the nearest health facility. As the negative binomial model is the nonlinear model, the linear approximation to the nonlinear model is made by estimating the partial effects evaluated at the means [27]. The regression model is given by Equation 2 [29]:

(2) $$y_{i}=\alpha +\sum_{j}\beta_{j}^{m}x_{\mathrm{ji}}+\sum_{k}\gamma_{k}^{n}z_{\mathrm{ki}}+\epsilon_{i}$$

where *y*_*i*_ is health services utilisation (i.e. outpatient visits or inpatient visits), $\beta_{j}^{m}$ and $\gamma_{k}^{n}$ are partial effects (i.e. *dy/dx*_*j*_ and *dy/dz*_*k*_) of each variable and evaluated at sample means; *ϵ*_*i*_ is the error term. The decomposition of the concentration index C could be specified as:

(3) $$C=\sum_{j}\left(\beta_{j}^{m}{\bar{x}}_{j}/\bar{y}\right)C_{j}+\sum_{k}\left(\gamma_{k}^{n}{\bar{z}}_{k}/\bar{y}\right)C_{k}+GC_{u}/\bar{y}$$

Where $\bar{y}$ is the mean of y, *C*_*j*_ and *C*_*k*_ are the concentration indexes for *x*_*j*_ and *z*_*k*_, $\bar{x_{j}}$ and ${\bar{z}}_{k}$ are the means of *x*_*j*_ and *z*_*k*_. The first term on the right side of Equation 3 denotes the contribution of need variables to inequality, the second denotes the contributions of control variables, the last term is the generalised concentration index of *ϵ*_*i*_. All analyses were performed in Stata version 12.1 (StataCorp LP, College Station, Texas, USA).

## Results

### Matching

Logistic regression models were used to estimate the propensity scores of the insured and uninsured residents for UEBMI and URBMI respectively in this study. After one-to-one matching, the mean propensity scores of the UEBMI insured residents and uninsured residents are 0.638 and 0.639, and the mean propensity scores of the URBMI insured residents and uninsured residents are 0.411 and 0.413; both differences are statistically insignificant. Tables 3 and 4 further show that, after matching most of standardised biases in UEBMI and URBMI below 5%, all the p values are larger than 0.05, indicating good matching performance. Based upon a one-to-one matching analysis, two new databases consisting of matched residents were produced for the two insurance schemes. The first data set contains 507 UEBMI insured residents and 507 uninsured residents, whilst the second data set contains 769 URBMI insured residents and 769 uninsured residents. These two data sets were used to further study the health utilisation equity.

**Table 3:** The difference in covariate variables for the UEBMI insured residents and uninsured residents before and after the matching (%)

	**Before matching**				**After matching**			
	**Insured**	**Uninsured**	**Standardized bias (%)**	**p**	**Insured**	**Uninsured**	**Standardized bias (%)**	**p**
Annual personal expenditure	8.94	8.64	54.8	0.000	8.76	8.74	4.3	0.490
Female	46.3	57.3	-22.1	0.000	47.3	50.5	-6.4	0.315
45-59	41.3	22.9	40.3	0.000	34.1	36.7	-5.6	0.394
60+	26.0	16.7	22.7	0.000	16.8	15.0	4.4	0.440
Health status	4.34	4.38	-20.5	0.000	4.36	4.36	-2.2	0.729
Married	87.5	64.0	57.1	0.000	82.6	81.9	1.9	0.743
Other marital status	7.8	10.6	-9.9	0.003	8.5	8.3	0.7	0.910
Primary school	9.2	10.5	-4.6	0.169	10.1	11.4	-4.6	0.478
Junior middle school	26.5	31.4	-10.7	0.001	32.2	30.8	3.0	0.636
Senior middle school	39.2	34.4	9.9	0.004	38.9	39.1	-0.4	0.949
Diploma	13.9	7.4	21.2	0.000	10.1	10.3	-0.6	0.917
College degree or above	8.0	7.4	2.3	0.500	4.9	5.1	-0.7	0.886
Retired	41.8	7.8	85.5	0.000	20.7	19.5	3.0	0.639
Student	0.1	14.7	-58.3	0.000	0.2	0.2	0.0	1.000
Unemployed	7.9	57.1	-123.4	0.000	33.9	31.6	5.9	0.422
Guanzhong	87.6	82.8	13.4	0.000	85.2	86.2	-2.8	0.654
Shannan	6.0	8.4	-9.0	0.006	7.1	7.1	0.0	1.000
Commercial health insurance	5.8	4.3	7.1	0.041	5.1	4.3	3.6	0.555
Household population	1.10	1.19	-25.3	0.000	1.18	1.19	-2.4	0.713

**Table 4:** The difference in covariate variables for the URBMI insured residents and uninsured residents before and after the matching (%)

	**Before matching**				**After matching**			
	**Insured**	**Uninsured**	**Standardized bias (%)**	**p**	**Insured**	**Uninsured**	**Standardized bias (%)**	**p**
Annual personal expenditure	8.72	8.65	12.0	0.010	8.71	8.72	-1.3	0.783
Female	58.2	56.6	3.2	0.480	58.5	58.5	0.0	1.000
45-59	23.3	21.8	3.6	0.432	23.5	23.0	1.2	0.809
60+	16.4	15.4	2.9	0.520	16.4	16.5	-0.4	0.945
Health status	4.39	4.39	-1.1	0.810	4.38	4.38	0.0	0.999
Married	63.2	61.5	3.4	0.453	63.5	61.5	4.0	0.430
Other marital status	11.0	10.4	1.9	0.681	10.8	11.6	-2.5	0.628
Primary school	8.2	9.4	-4.4	0.340	8.3	7.2	4.1	0.391
Junior middle school	27.9	31.7	-8.3	0.070	28.5	29.9	-3.1	0.538
Senior middle school	43.2	35.7	15.3	0.001	42.4	42.4	0.0	1.000
Diploma	8.7	7.3	5.1	0.257	8.5	9.0	-1.9	0.718
College degree or above	4.1	7.2	-13.5	0.004	4.2	3.6	2.3	0.599
Retired	7.6	7.8	-0.4	0.923	7.8	8.6	-2.9	0.577
Student	13.9	17.6	-10.1	0.029	14.2	14.6	-1.1	0.828
Unemployed	51.3	55.1	-7.5	0.102	52.1	51.4	1.6	0.760
Guanzhong	95.7	89.5	23.6	0.000	95.6	95.8	-1.0	0.801
Shannan	0.0	0.5	-9.9	0.049	0.0	0.0	0.0	1.000
Commercial health insurance	4.6	4.2	1.6	0.718	4.7	4.0	3.2	0.533
Household population	1.17	1.20	-7.8	0.086	1.18	1.19	-2.1	0.691

### Measuring the equity of health service utilisation

Negative binomial models were used to decompose the inequality of outpatient and inpatient utilisation. All relevant variables in the analysis are described in Table 5. It could be seen that the insured residents used more outpatient and inpatient health services than the uninsured.

**Table 5:** Description of dependent variables, need variables and control variables in negative binomial models (mean/percent)

	**UEBMI**		**URBMI**	
	**Insured**	**Uninsured**	**Insured**	**Uninsured**
Dependent variable				
Outpatient visit rate (%)	13.02	7.69	12.74	7.93
Inpatient visit rate (%)	6.11	5.52	4.68	2.99
Need variables				
Female	47.3	50.5	58.5	58.5
45-59	34.1	36.7	23.5	23.0
60+	16.8	15.0	16.4	16.5
Health status	79.2	80.2	81.5	81.9
Illness	12.9	12.8	12.3	10.2
Chronic disease	16.6	17.0	18.7	16.0
Control variables				
Annual personal expenditure	7192	7322	6840	7026
Married	82.6	81.9	63.5	61.5
Other married status	8.5	8.3	10.8	11.6
Commercial health insurance	5.1	4.3	4.7	4.0
Household population	3.5	3.6	3.5	3.5
Distance to the nearest health institution	10.0	8.8	9.8	9.5

Table 6 shows the estimated partial coefficients of independent variables in the negative binomial models, which will be employed to decompose the concentration index of health service utilisation by using Equation 3.

**Table 6:** The estimated partial coefficients in the negative binomial models

	**UEBMI**				**URBMI**			
	**Outpatient visits**		**Inpatient visits**		**Outpatient visits**		**Inpatient visits**	
	**Insured**	**Uninsured**	**Insured**	**Uninsured**	**Insured**	**Uninsured**	**Insured**	**Uninsured**
Female	0.038 (0.038)	-0.044* (0.026)	0.007 (0.009)	0.007 (0.013)	<0.001 (0.015)	-0.033 (0.022)	0.017 (0.013)	0.011 (0.007)
45-59	0.058 (0.050)	-0.040* (0.023)	0.002 (0.011)	-0.023 (0.015)	0.034 (0.028)	-0.003 (0.022)	-0.004 (0.015)	-0.011 (0.007)
60+	-0.044 (0.038)	0.025 (0.041)	-0.001 (0.010)	0.003 (0.018)	0.006 (0.024)	0.015 (0.033)	0.002 (0.019)	0.000 (0.010)
Health status	0.071 (0.100)	-0.051 (0.056)	-0.020 (0.016)	-0.030 (0.020)	-0.114*** (0.044)	-0.060 (0.042)	-0.039 (0.025)	-0.013 (0.013)
Illness	—	—	0.022 (0.016)	0.027 (0.025)	—	—	0.018 (0.022)	0.030 (0.024)
Chronic disease	0.288* (0.167)	0.061 (0.058)	0.167*** (0.059)	0.063 (0.038)	0.149 (0.060)	0.139 (0.087)	0.034 (0.025)	0.022 (0.021)
Annual personal expenditure	-0.011 (0.038)	-0.010 (0.017)	0.010 (0.008)	0.022* (0.012)	-0.004 (0.016)	-0.014 (0.015)	0.023* (0.012)	0.015** (0.007)
Married	-0.112 (0.118)	0.020 (0.027)	-0.020 (0.027)	0.023** (0.012)	0.019 (0.025)	0.005 (0.024)	0.007 (0.017)	0.002 (0.008)
Other married status	0.009 (0.085)	0.001 (0.053)	0.001 (0.018)	—	0.008 (0.038)	-0.004 (0.032)	-0.014 (0.019)	-0.010 (0.007)
Commercial health insurance	0.501 (0.536)	—	-0.006 (0.015)	0.081 (0.074)	0.281 (0.196)	0.011 (0.061)	-0.009 (0.021)	0.028 (0.033)
Household population	-0.078 (0.057)	-0.053* (0.031)	0.010 (0.010)	-0.022 (0.015)	-0.036* (0.018)	-0.051** (0.024)	-0.018 (0.015)	0.000 (0.009)
Distance to the nearest health institution	0.055* (0.034)	0.044** (0.021)	0.005 (0.007)	0.015 (0.011)	0.028** (0.012)	0.021 (0.016)	0.000 (0.009)	-0.006 (0.006)
LR	24.7	20.51	56.77	30.64	85.95	34.47	27.57	32.62
P	0.010	0.025	<0.001	0.001	<0.001	<0.001	0.006	0.001

Notes: Standard errors of partial coefficients are in the parentheses. *Significant at 10%, **Significant at 5%, and ***Significant at 1%. “—” means the variables are not included in the models.

Table 7 shows the contributions of each independent variable on the inequality in health service utilisation. By subtracting the contribution of need variables from the concentration index of health service utilisation, we could get the horizontal inequity index of health service utilisation. Table 8 shows that the horizontal inequity indexes of outpatient utilisation for the UEBMI insured and uninsured residents are 0.1256 and -0.0511, which means there is a pro-rich inequity of outpatient utilisation for the people covered by UEBMI (i.e. with the same need for outpatient service, the rich people utilise more outpatient services than the poor people) and pro-poor inequity of outpatient utilisation for the uninsured people. Differing from UEBMI, with the reported horizontal inequity indexes of -0.1593 and 0.0967, the inequity of outpatient utilisation for the URBMI insured residents and uninsured residents are pro-poor and pro-rich, respectively.

**Table 7:** The contributions of each independent variable on the inequality in health service utilisation

	**UEBMI**				**URBMI**			
	**Outpatient visits**		**Inpatient visits**		**Outpatient visits**		**Inpatient visits**	
	**Insured**	**Uninsured**	**Insured**	**Uninsured**	**Insured**	**Uninsured**	**Insured**	**Uninsured**
Female	0.0084 (6.8)	-0.0237 (38.5)	0.0033 (2.4)	0.0050 (1.7)	0.0000 (0.0)	0.0000 (0.0)	0.0010 (0.6)	0.0000 (0.0)
45-59	-0.0139 (-11.4)	0.0057 (-9.2)	-0.0009 (-0.7)	0.0046 (1.6)	-0.0021 (1.0)	0.0003 (0.3)	0.0006 (0.4)	0.0030 (0.9)
60+	-0.0077 (-6.3)	-0.0038 (6.1)	-0.0003 (-0.2)	-0.0006 (-0.2)	-0.0012 (0.6)	-0.0027 (-3.1)	-0.0013 (-0.8)	0.0002 (0.1)
Health status	0.0009 (0.7)	0.0024 (-3.9)	-0.0005 (-0.4)	0.0019 (0.7)	-0.0089 (4.3)	-0.0045 (-5.0)	-0.0083 (-5.2)	-0.0026 (-0.8)
Illness	—	—	0.0020 (1.5)	-0.0012 (-0.4)	—	—	-0.0036 (-2.3)	-0.0050 (-1.6)
Chronic disease	0.0093 (7.6)	0.0090 (-14.6)	0.0115 (8.4)	0.0128 (4.3)	-0.0367 (17.6)	-0.0004 (-0.4)	-0.0225 (-14.2)	-0.0002 (-0.1)
Annual personal expenditure	-0.0237 (-19.3)	-0.0412 (67.0)	0.0437 (31.9)	0.1292 (43.5)	-0.0088 (4.2)	-0.0511 (-57.2)	0.1353 (85.1)	0.1490 (47.2)
Married	-0.0100 (-8.2)	-0.0043 (7.0)	-0.0039 (-2.8)	-0.0070 (-2.4)	0.0018 (-0.9)	-0.0010 (-1.1)	0.0019 (1.2)	-0.0010 (-0.3)
Other married status	0.0000 (0.0)	0.0000 (-0.1)	0.0000 (0.0)	—	-0.0013 (0.6)	0.0003 (0.3)	0.0058 (3.6)	0.0017 (0.5)
Commercial health insurance	0.0265 (21.6)	—	-0.0007 (-0.5)	0.0184 (6.2)	0.0309 (-14.8)	0.0012 (1.3)	-0.0026 (-1.6)	0.0082 (2.6)
Household population	0.0264 (21.6)	0.0297 (-48.3)	-0.0075 (-5.5)	0.0171 (5.8)	-0.0032 (1.5)	0.0162 (18.1)	-0.0044 (-2.8)	0.0004 (0.1)
Distance to the nearest health institution	0.0030 (2.4)	-0.0240 (39.0)	0.0006 (0.4)	-0.0109 (-3.7)	-0.0061 (2.9)	-0.0088 (-9.8)	-0.0003 (-0.2)	0.0063 (2.0)

Note: The proportions of each variable’s contribution in the concentration index of health service utilisation are in the parentheses.

**Table 8:** Horizontal inequity index of health service utilisation

	**UEBMI**		**URBMI**	
	**Outpatient utilisation**	**Inpatient utilisation**	**Outpatient utilisation**	**Inpatient utilisation**
**Insured**				
CI	0.1225	0.1372	-0.2082	0.1590
Contribution of health need	-0.0031	0.0150	-0.0489	-0.0341
**HI index**	0.1256	0.1222	-0.1593	0.1931
	(0.0249, 0.3230)	(0.0154, 0.3315)	(-0.2626, 0.0432)	(0.0887, 0.3977)
**Uninsured**				
CI	-0.0615	0.2971	0.0894	0.3153
Contribution of health need	-0.0104	0.0225	-0.0073	-0.0046
**HI index**	-0.0511	0.2746	0.0967	0.3199
	(-0.0833, 0.0120)	(0.1358, 0.5467)	(0.0486, 0.1910)	(0.1655, 0.6225)

Notes: CI - concentration index; HI - horizontal inequity. 95% confidence intervals of HI index are in the parentheses.

All the horizontal inequity indexes for inpatient health services are positive, suggesting a pro-rich horizontal inequity for both insured and uninsured residents. In addition, the horizontal inequity indexes of inpatient utilisation for the uninsured are larger than the insured, indicating a larger inpatient utilisation inequity for the uninsured.

## Discussion

Using the cross-sectional data from the 4th National Health Services Survey and an extended survey sample in Shaanxi Province, this study studied the effects of UEBMI and URBMI on the equity of outpatient and inpatient utilisation. To partly solve the potential issue of adverse selection the PSM method was employed to generate a matched study sample before analysing the inequality of residents’ health service utilisation. Using the one-to-one matching algorithm, the standardised bias and *t*-test of covariates both suggest a good matching performance, that the insured and uninsured are comparable on the observable characteristics after the matching exercise.

The horizontal equity result of outpatient utilisation for the UEBMI matched sample (pro-rich inequity for UEBMI enrollees and pro-poor for the matched uninsured) suggests that as part of the reimbursement policy of UEBMI, the policy of MSAs reduced the outpatient utilisation equity. For the UEBMI scheme, the value of medical saving accounts is proportionate to the individual’s income; therefore, higher income means more value in the outpatient account. As the MSA account can only be used to pay for the outpatient service, high-income residents have the incentive to overuse the outpatient service. Thus, the gap of outpatient utilisation between the high-income and low-income residents would be enlarged and the equity of outpatient utilisation would be reduced. This result is consistent with those reported by Liu et al. and Dong [11,12].

For inpatient utilisation, the horizontal inequity index was reduced to 55.5% by UEBMI, which means the implementation of UEBMI made the equity of inpatient utilisation improve significantly. According to the reimbursement policy, a proportion of the insured residents’ inpatient expense was reimbursed by UEBMI, which means the insured residents were facing a decreased price for inpatient services. The demand for inpatient services thus increased [30]. As the low-income residents are more sensitive to the change of inpatient prices than the high-income residents, when the inpatient price decreased, the low-income residents’ demand for inpatient services would increase much more than the high-income residents. Therefore, the equity of inpatient utilisation would be improved. Even so, the pro-rich inequity of inpatient utilisation was still observed for the insured residents in this study, suggesting the UEBMI policy needs to be further improved.

The horizontal equity result of outpatient utilisation from the URBMI matched sample is opposite to what has been found based on UEBMI matched samples (i.e. pro-poor inequality for URBMI enrollees and pro-rich for the matched uninsured). Differing from the UEBMI, the MSA was not established in URBMI in 2008. The critical (i.e. chronic or fatal disease) outpatient utilisation was reimbursed through the social pooling funds. According to this design, both of the high-income and low-income residents’ outpatient utilisation could be increased. Since the low-income individuals were more sensitive to price changes, the low-income residents would utilise more outpatient services than the high-income residents.

For inpatient utilisation, the horizontal inequity index was reduced by 39.6% by URBMI, which means URBMI improved the insured residents’ inpatient utilisation significantly. This is the same as the UEBMI, since the URBMI enrollees were facing a lower price for inpatient health services, they had the incentive to increase the demand for inpatient services, and since the poor are more sensitive to price change, the gap between low-income and high-income residents could be reduced. In addition, we found that the horizontal inequity of inpatient utilisation was reduced less by URBMI than by UEBMI, which may relate to the fact that in the benefit package design, the inpatient reimbursement rate is lower for the URBMI scheme than for the UEBMI scheme.

It is important to bear in mind that there are some limitations in this study. Firstly, a larger sample size would be ideal for assessing the equity of inpatient services. In this study, we generated two groups of comparable sub-samples (insured vs. uninsured) for analysis using PSM: to serve this aim, the incomparable sample was later dropped and this lead to a decreased sample size. This is a trade-off between sample size and comparability and we believe the latter matters the most. The final sample size of this study is similar to previously published studies [14,31]. Secondly, although the insured and uninsured residents have been matched before comparing their health utilisation equity, since this is not an experimental study, we cannot control the unobservable characteristics (such as risk attitude and personality), the casual relationship cannot be attained by this study. Considering URBMI is a voluntary scheme and UEBMI is a compulsory scheme, the unobservable heterogeneity would be more likely to impact on the results on URBMI. Thirdly, this study used a household survey data from 2008. Ever since then the UEBMI policy remains the same in Shaanxi Province, whilst the URBMI policy has changed recently. A key difference for the URBMI policy is the introduction of the MSA for outpatient service utilisation in 2012. The relevant results reported in this study may not reflect the insurance effect of the latest insurance policy changes. The future study should further empirically study the effect of MSA on the equity of outpatient health service utilisation for the URBMI enrollees. Lastly, so far only the horizontal equities of health service utilisation were investigated in this study. The vertical equities of health service utilisation should be conducted in a future study.

## Conclusion

The implementation of UEBMI increased the pro-rich inequity of outpatient service utilisation, whilst the URBMI increased the pro-poor inequity of outpatient service utilisation in Shaanxi Province, China. Both UEBMI and URBMI reduced the pro-rich inequity of inpatient service utilisation.

## Competing interests

There are no competing interests for any of the authors.

## Authors’ contribution

ZL Zhou, LZ, and JG conceived and designed the study. ZL Zhou and GC analyzed the data. LZ and JG acquired data. ZL Zhou, GC and ZY Zhou wrote the paper. GC, JG, ZL Zhou and ZY Zhou critically revised the manuscript. All authors read and approved the final manuscript.

## Authors’ information

Zhongliang Zhou and Liang Zhu are co-first authors.

## Supplementary Material

Additional file 1: Table S1 Sample representative tests. Click here for file
