# Supplementary material for: The effects of China’s urban basic medical insurance schemes on the equity of health service utilisation: evidence from Shaanxi Province
Source: Int J Equity Health. 2014 Mar 9;13:23. doi: 10.1186/1475-9276-13-23 (PMC4016277; doi:10.1186/1475-9276-13-23)
Supplement: Additional file 1: Table S1 — Sample representative tests. [file 1475-9276-13-23-S1.doc]

**Additional file 1: Table S**1. Sample representative tests

|  | Myer’s Index | Delta Missimilarity Index | Gini Concentration Ratio |
| --- | --- | --- | --- |
| Urban & Rural residents | 1.67 | <0.0001 | −0.0028 |
| Urban residents | 1.79 | <0.0001 | 0.0523 |
| Rural residents | 2.16 | <0.0001 | 0.0374 |

Note: Delta Missimilarity Index and Gini Concentration Ratio were calculated based on the data of fifth census in 2000.
